# Thoracoscopic Right Lower Lobectomy in a Patient With a Common Trunk of the Right Pulmonary Veins

**DOI:** 10.1016/j.atssr.2022.10.011

**Published:** 2022-10-29

**Authors:** Daiki Noda, Shintaro Yokoyama, Sho Setojima, Shiro Kibe, Kunihiro Ozaki, Ryozo Hayashida, Yutaka Nishimura, Atsushi Osoegawa, Masahiro Mitsuoka, Kenji Sugio

**Affiliations:** 1 Department of Surgery, Oita Prefecture Saiseikai Hita Hospital, Hita, Japan; 2 Department of Thoracic and Breast Surgery, Oita University Faculty of Medicine, Yufu, Japan; 3 Department of Surgery, Kurume University School of Medicine, Kurume, Japan

## Abstract

Varied branching patterns of the pulmonary veins are widely known; however, the common trunk of the right pulmonary veins is sparsely described. We report the details of a right lower lobectomy in a patient with a common trunk of the right pulmonary veins. Besides an attentive interpretation of computed tomography images, 3-dimensional reconstruction aids in the preoperative recognition of this anomaly. To ensure safe division of the lower lobe vein, adequate exposure of the pulmonary hilum is needed, supported by prior interlobar fissure division between the middle and lower lobes.

Divergence in the anatomy of the pulmonary veins is well known to vary and is critical during anatomic lung resections. The development of computed tomography (CT), including 3-dimensional visualization, has allowed us to precisely define anatomic variations in the pulmonary vessels preoperatively. Thoracic surgeons should routinely be aware that unexpected intraoperative bleeding may occur when rare branching patterns of the pulmonary vessels are misrecognized. The common trunk of the left pulmonary veins is a dangerous branching form because its misidentification leads to fatal complications during and after anatomic lung resection; however, that of the right pulmonary veins is extremely rare and has not been adequately reported. Herein, we describe the detailed clinical findings and thoracoscopic procedures of a right lower lobectomy in a lung cancer patient with a common trunk of the right pulmonary veins.

A 68-year-old woman with lung adenocarcinoma was scheduled for a right lower lobectomy at Oita Prefecture Saiseikai Hita Hospital. Preoperative CT revealed a 16-mm nodule at S6 of the right lung (Figure 1A). Because the ipsilateral interlobar lymph node (No. 11) was significantly swollen, she was clinically diagnosed with stage 2B (T2a N1 M0). A common trunk of the right pulmonary veins was suspected on the basis of the CT findings (Figures 1B, 1C). Three-dimensional visualization using Synapse Vincent software (Fuji Film Medical) helped identify this stereoscopically (Figure 1D). No anatomic aberrations were observed in the pulmonary arteries or bronchi.

**Figure 1:**
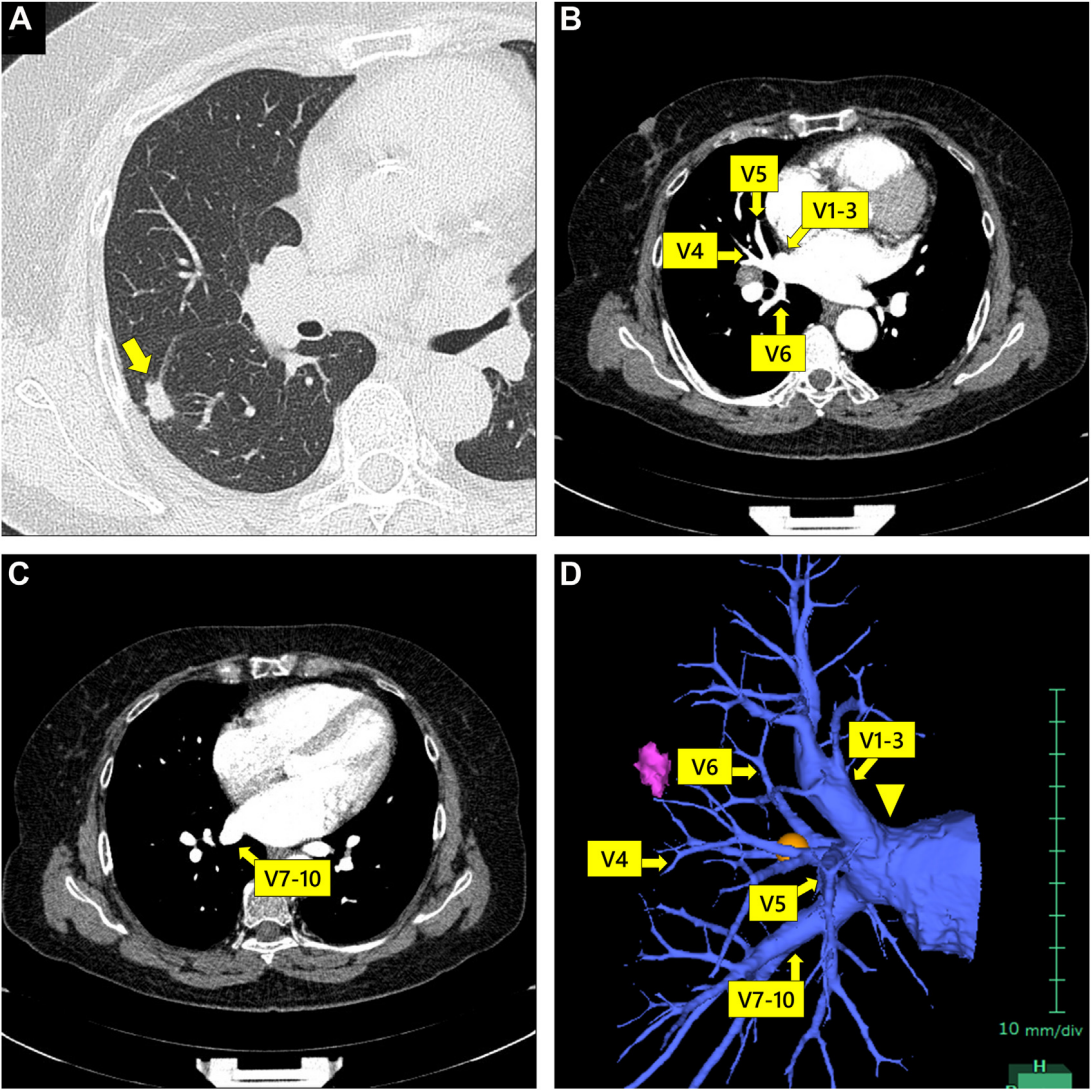
Preoperative computed tomography images. (A) The tumor located at S6 of the right lung (arrow). (B, C) Sequential images demonstrate the common trunk of the right pulmonary veins. (D) Three-dimensional imaging provides stereoscopic visualization of the common trunk of the right pulmonary veins (arrowhead).

Video-assisted thoracoscopic surgery with 3 access ports was performed. The caudal border of the lower lobe vein was located more cranially than usual in the pulmonary hilum. The cranial rim of V6 was hidden by the middle lobe vein, which was not observed on the ventral side (Figure 2A). The bifurcation between the middle and lower lobe veins as well as the upper and middle lobe veins was confirmed in the pleural cavity, not in the pericardial space. Although the posterior aspect allowed us to dissect the roof of V6, the cranial space over V6 was too confined to encircle the lower lobe vein. After the lower lobe artery was divided and interlobar fissure division was performed between the middle and lower lobes, the V6 was circumferentially exposed. After hilar lymph node dissection, the lower lobe vein and bronchus were divided by mechanical staplers, and a right lower lobectomy was performed (Figure 2B). Subsequently, mediastinal lymph node dissection was performed. Contrast-enhanced CT on the fifth postoperative day revealed no thrombosis at the stump of the pulmonary vein. The postoperative course was uneventful. The tumor was histologically diagnosed as a papillary adenocarcinoma with a 15-mm diameter. The pathologic stage was 3A (T1b N2 M0) with subcarinal lymph node (No. 7) metastasis.

**Figure 2:**
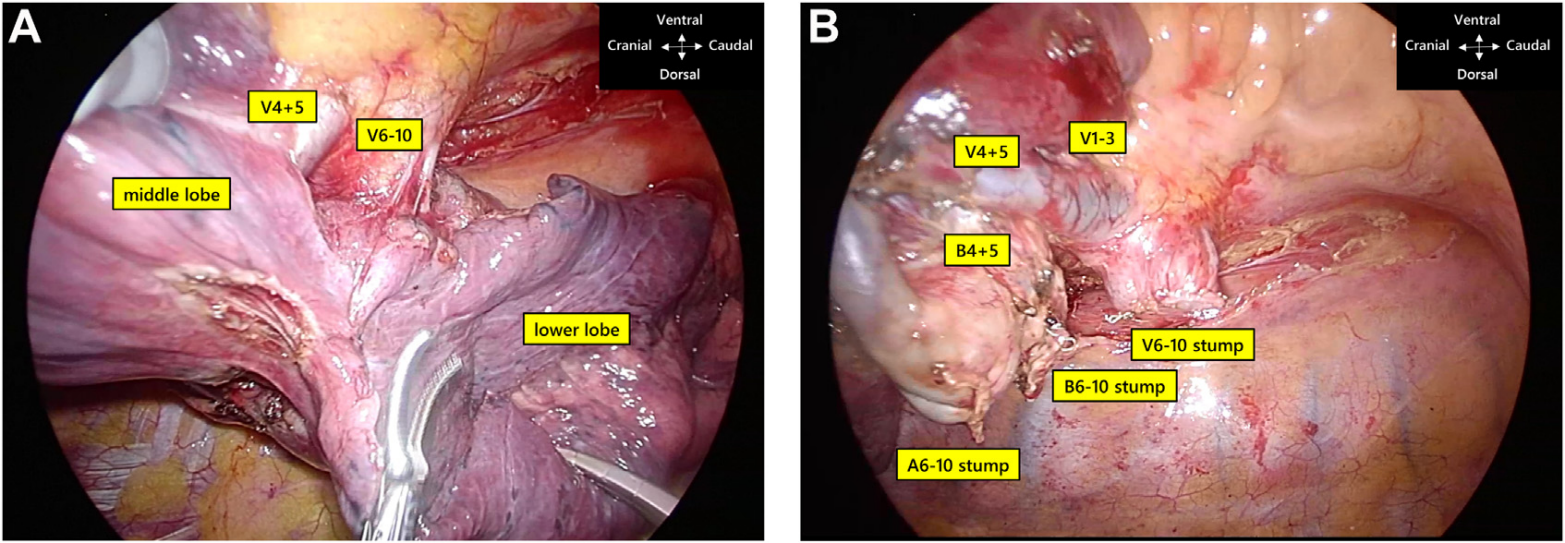
Intraoperative findings. (A) The cranial edge of V6 behind the middle lobe vein could not be identified from the ventral aspect. (B) After interlobar fissure division between the middle and lower lobes, the lower lobe vein could be circumferentially exposed and divided.

## Comment

The recent increase in pulmonary segmentectomy emphasizes the importance of the branching pattern of the pulmonary veins, which determines the pulmonary segmental anatomy. Venous branches generally occur on the right side as the 2 upper and lower lobe veins, with the middle lobe vein flowing into the upper lobe vein, and on the left side as the 2 upper and lower lobe veins. However, Polaczek and coworkers^1^ reported this typical pattern in only 27.4% of cases based on CT surveillance.

The surgical procedures for right lower lobectomy are deeply affected by venous anomalies. First, V2, which runs a retrobronchial course joining the inferior pulmonary vein, is sometimes encountered (6.7% of patients).^1^ In such cases, the lower lobe veins should be carefully divided to ensure V2 venous drainage. Moreover, Endo and colleagues^2^ reported dangerous venous drainage not only in V2 but also in an aggregate upper lobe vein flowing into the inferior pulmonary vein. Second, the middle lobe vein streaming into the inferior pulmonary vein should be noted as it occurs in 3.0% to 7.1% of cases.^1^^,^^3^ This pattern carries the risk of middle lobe dysfunction after right lower lobectomy due to congestion when the middle lobe vein is mistakenly incised along with the lower lobe vein. Finally, as shown in our case, the common trunk of the right pulmonary veins should be acknowledged. Compared with that of the left pulmonary veins, which occurs in 11.1% to 33.1% of cases, the common trunk of the right pulmonary veins is confirmed in only 0.7% of cases, indicating that it may not be sufficiently recognized by thoracic surgeons.^1^^,^^4^ Careful surgical strategies are required for patients with this aberration. The cranial edge of the common trunk is caudally located in the pulmonary hilum. Venous reconstruction of the upper and middle lobe veins is required when the common trunk is misidentified as the lower lobe vein. Sufficient exposure of the hilar structures is important, as reported in the case of left upper lobectomy in a patient with left common pulmonary veins.^5^ Furthermore, division of the fused fissures between the middle and lower lobes before the lower lobe vein incision should contribute to achieving this goal. Yamada and coworkers^6^ demonstrated the usefulness of a similar technique in patients with left common pulmonary veins.

This report describes both CT images and intraoperative findings of a patient with a common trunk of the right pulmonary veins. Preoperative recognition based on careful interpretation of preoperative CT scans and efficacious surgical procedures enable successful right lower lobectomy in patients with a common trunk of the right pulmonary veins.
